# WOmen's Action for Mums and Bubs (WOMB) Trial Protocol: A Non-randomized Stepped Wedge Implementation Trial of Participatory Women's Groups to Improve the Health of Aboriginal and Torres Strait Islander Mothers and Children in Australia

**DOI:** 10.3389/fpubh.2020.00073

**Published:** 2020-03-18

**Authors:** Karen Carlisle, Catrina Felton-Busch, Yvonne Cadet-James, Judy Taylor, Ross Bailie, Jane Farmer, Megan Passey, Veronica Matthews, Emily Callander, Rebecca Evans, Janet Kelly, Robyn Preston, Michelle Redman-MacLaren, Haylee Fox, Adrian Esterman, Merrick Zwarenstein, Sarah Larkins

**Affiliations:** ^1^College of Medicine and Dentistry, James Cook University, Douglas, QLD, Australia; ^2^Anton Breinl Research Centre for Health Systems Strengthening, James Cook University, Townsville, QLD, Australia; ^3^Australian Institute of Tropical Health and Medicine, James Cook University, Townsville, QLD, Australia; ^4^Centre for Rural and Remote Health, James Cook University, Mount Isa, QLD, Australia; ^5^Indigenous Education and Research Centre, James Cook University, Douglas, QLD, Australia; ^6^University Centre for Rural Health, University of Sydney, Lismore, NSW, Australia; ^7^Social Innovation Research Unit, Swinburne University of Technology, Hawthorn, VIC, Australia; ^8^School of Medicine, Griffith University, Gold Coast, QLD, Australia; ^9^Adelaide Nursing School, University of Adelaide, Adelaide, SA, Australia; ^10^School of Health, Medical and Applied Science, CQUniversity, Townsville, QLD, Australia; ^11^College of Medicine and Dentistry, James Cook University, Smithfied, QLD, Australia; ^12^Australian Institute of Tropical Health and Medicine, James Cook University, Smithfield, QLD, Australia; ^13^Centre for Studies in Family Medicine, Department of Family Medicine and Schulich School of Medicine & Dentistry, Western Centre for Public Health and Family Medicine, Western University, London, ON, Canada

**Keywords:** indigenous health, complex intervention, cluster stepped wedge, community participation, maternal and child health, empowerment

## Abstract

**Introduction:** In Australia, there have been improvements in Aboriginal and Torres Strait Islander maternal health, however inequities remain. There is increasing international evidence illustrating the effectiveness of Participatory Women's Groups (PWGs) in improving Maternal and Child Health (MCH) outcomes. Using a non-randomized, cluster stepped-wedge implementation of a complex intervention with mixed methods evaluation, this study aims to test the effectiveness of PWGs in improving MCH within Indigenous primary care settings in Australia and how they operate in various contexts.

**Methods:** This study takes place in ten primary health care services across Australia and involves the recruitment of existing PWGs or the setting up of new PWGs. Services are paired based on geography for practical reasons and two services commence the PWG intervention at three monthly intervals, with the initial four services being those with existing women's groups. Implementation of the PWGs as an intervention involves training local facilitators of PWG groups, supported engagement with local MCH data through workshops, PWGs identifying and prioritizing issues and strengths and co-implementing solutions with health services. Outcomes are measured with yearly MCH audits, a cost-effectiveness study, and process evaluation of community participation and empowerment.

**Discussion:** This study is the first to formally implement and quantitatively, yet with contextual awareness, measure the effect of applying a community participation intervention to improve the quality of Aboriginal and Torres Strait Islander MCH in Australia. Findings from this work, including detailed theory-producing qualitative analysis, will produce new knowledge of how to facilitate improved quality of MCH care in Indigenous PHC settings and how to best engage community in driving health care improvements.

**Trial registration:** Australian New Zealand Clinical Trials Registry (ANZCTR): ACTRN12618000945224.

Web address: http://www.ANZCTR.org.au/ACTRN12618000945224.aspx

## Introduction

The health, social and economic outcomes for Aboriginal and Torres Strait Islander Australians continue to be substantially worse than for other Australians (1–3). In Australia, improving the quality and consistency of PHC provided to Aboriginal peoples and Torres Strait Islanders is an essential part of the Australian Government's Close the Gap program (4). Maternal and child health (MCH) is consistently named a priority area by Aboriginal and Torres Strait Islander peoples, policy makers health service providers, and researchers (5, 6). Although Aboriginal and Torres Strait Islander infant mortality rates and the proportion of low birth weight babies born are falling, they remain almost double those of non-Indigenous infants (1).

Internationally, primary health care (PHC) is recognized as critical to addressing inequities in health status (2, 3). While Indigenous PHC services [both Aboriginal Community Controlled Health Services (ACCHS) and government services] provide PHC for Indigenous people, the quality of care and health outcomes can vary significantly between services (3, 7).

Continuous Quality Improvement (CQI) aims to facilitate ongoing improvement in the quality of PHC by using objective information to analyze and improve systems, processes and outcomes (8). A CQI strategy based on audit and feedback cycles of “plan, do, study, act” provides a theoretical, coherent and practical way for PHC services to identify, address and overcome barriers to quality care (9, 10). Modern CQI approaches incorporating service user participation, engagement, shared-decision making and tailoring to context address both scientific and humanistic values (10). Such approaches may also offer the potential to enhance MCH through the improvements in quality made in the PHC setting. International empirical evidence suggests that an integrated approach that includes community women as active participants (11) working alongside service providers can improve both the quality and outcomes of MCH care in a relatively short time period (11–13). Furthermore, CQI fits with the principles and values of Aboriginal and Torres Strait Islander people (expressed in national statements on research and cultural respect) (14, 15). Despite the demonstrated benefits of community participation (16), very few quality improvement interventions in Aboriginal and Torres Strait Islander PHC have focused on factors beyond service provision as part of their implementation. None have explicitly tested community participation aimed at enhancing community decision-making and co-production in health care and health service design. Similarly, in evaluating quality improvement interventions, the cost effectiveness of community participation in light of MCH improvements in service provision and health outcomes has not been assessed. Hence, there is a need to add to the evidence in this area.

There is persuasive international research illustrating the effectiveness of participatory women's groups (PWGs) in improving MCH outcomes such as neonatal and maternal mortality rates via improved quality of care, women's empowerment, and new learning (11, 16–18). There is considerable evidence internationally (19, 20) and in Australian Aboriginal and Torres Strait Islander settings (21) that empowerment of women is achieved through participation in such women's groups. Empowerment means healing from past wounds, developing strength and skills to live life in a positive way, to have good relationships with others and to work together to make communities a better place (22). A systematic review with meta-synthesis found that participatory interventions using women's groups are effective in improving neonatal and maternal survival (23). This led to the identification of PWGs, coupled with health systems strengthening and community action, as highly effective strategies for improving MCH that are low-cost, sustainable, and scalable.

Morrison et al. (20) have named four mechanisms to explain the positive impact of women's groups on health outcomes in a study of maternal and newborn health in Nepal. The groups: (a) learn about health; (b) develop confidence; (c) disseminate information in their communities; and (d) build community capacity for action (20). Based on this evidence, we hypothesize that improvements in MCH related to participation in PWGs may be mediated through measurable changes in empowerment.

The study discussed above took place in Nepal, with contextual factors including a weak health system funded through significant contributions from external development partners who are influential in decision-making. In addition, similar to most low-resource settings, the PHC system is dependent on extensive contributions from female community health volunteers (over 50,000 in Nepal). External agents call on these women volunteers to assist in conducting MCH interventions (24). By comparison, the Australian public health system is relatively strong and does not rely on volunteers to provide PHC services. Interventions are usually provided by health professionals, many of whom are unfamiliar with working collaboratively with women's groups. Yet, we know that working partnerships in Indigenous health are fundamental (25); PHC staff and managers need to be supported to understand the importance of community participation in co-producing responsive MCH services that aim to improve patient outcomes.

There are compelling examples of Aboriginal and Torres Strait Islander community responses to health and well-being needs that arise spontaneously within communities. The overarching example is the Aboriginal community controlled health movement as the expression of Indigenous cultural values within a predominantly western health sector (26). On a smaller scale, the Strong Women, Strong Babies, Strong Culture program in the Northern Territories employed older community women to work individually with pregnant women to improve health behavior and health care access. A small, but significant increase in birthweight over an 8 year period was demonstrated through this program (27). The Aboriginal-developed Family Wellbeing Program (FWB) is another empowerment-based participatory approach, designed to support the capacity for individuals, organizations and communities to gain control over their lives to improve wellbeing and quality of life (21, 28–30). FWB has been successfully applied by our team to MCH care, organizational change and team strengthening (amongst others). Reported benefits of FWB include greater understanding and mutual respect between health staff, an enhanced capacity to work with others toward common goals, and improved access to health services and high quality service delivery (31, 32).

This study examines whether PWGs, as an intervention, improve the quality of Indigenous MCH care. More specifically, it will determine the effectiveness of PWGs in improving quality of care and intermediate health and well-being outcomes in MCH; the cost-effectiveness of PWGs for improving quality of MCH care; the degree to which the PWGs are associated with a change in empowerment measured using the global empowerment measure (GEM) scores for women involved (22); and the processes through which PWGs exert effects in various contexts.

## Research Question and Objectives

Do participatory women's groups (PWGs), as an intervention, improve the quality of maternal and child health (MCH) care in Australian Indigenous primary health care (PHC) services?

The objectives are to determine:

1. The effectiveness of PWGs in improving quality of care and intermediate outcomes in MCH;
2. The cost-effectiveness of PWGs for improving quality of MCH care;
3. The degree to which the PWGs are associated with a change in global empowerment measure (GEM) scores for participating women; and
4. How and why PWGs exert their effects in various contexts.

## Methods and Analysis

### Design

A stepped wedge cluster implementation (non-randomized) of a complex intervention was chosen for ethical and logistical reasons. It allows all participating PHC services to receive the PWG intervention in sequential order, where evidence of effectiveness can be assessed with pre- and post-intervention observations from each service (33). This design has an advantage in improving power with smaller numbers of services where intra-cluster correlation is high (optimizing feasibility). By allowing services to act as their own historical controls it also accounts for underlying differences between clusters that may confound results in the more traditional parallel cluster designs. Through the collection of routinely recorded MCH audit data, the study assesses the effectiveness of a complex intervention involving PWGs addressing issues in MCH care to improve outcomes amongst Indigenous mothers and babies. To complement the design, qualitative research with PHC services and PWGs will explore contextual barriers and enablers to successful implementation of the PWG in various settings throughout Australia.

Recognizing that patients, clinicians and policy makers need real world evidence to make informed decisions about their healthcare, the study represents a range of typical Indigenous PHC settings, using realistically attainable intervention approaches under real-world conditions, and reporting relevant outcomes to all stakeholders (34).

To test the effectiveness of the PWG intervention, this design is blended with our commitment to a participatory approach informed by Indigenous ways of being, knowing and doing and an all-teach and all-learn philosophy. This is done in part through optimizing the flexibility to respond to the rich and varying needs and contexts of individual health services and communities, whilst maintaining the integrity and consistency of the intervention. One example of this is that initial meetings with participating services determined that there are two groups of services, four services with existing functional women's groups and active auditing (early services) and six services who do not yet have a functional women's group and/or have auditing delays (later services). Recognition of these differences, and a rejection of the concept of randomization as disempowering by participating services and partners has led to a revision of the design in terms of randomization of the order of intervention, as described below. Furthermore, service representatives and PWG facilitators are all involved in collaborative planning and training meetings, steering group meetings, and team communication strategies throughout the life of the study. This study protocol was developed as required by the Standard Protocol Items: Recommendations for Interventional Trials (SPIRIT) (Supplementary Material 1).

### Study Setting

This study is conducted in partnership with ten *primarily Indigenous* PHC services in Queensland, New South Wales, Northern Territory and Western Australia. *Primarily Indigenous* refers to services that are an Aboriginal Community Controlled Health Service or government service funded primarily to service Aboriginal or Torres Strait Islander people. Participating services represent a range of jurisdictions, service size, remoteness, governance models and CQI tools.

### Study Participants

The target population to whom our findings apply is primarily Indigenous PHC services and women and children who access Indigenous PHC services. Thus, PHC services are eligible to participate if they fulfil the following inclusion criteria: (a) are a primarily Indigenous PHC service (b) are engaged in some auditing of MCH service provision using a recognized and evidence-based audit tool (35, 36); (c) have a formal or informal community women's group associated with the service or a desire to establish one; (d) are willing to support at least one staff member to attend facilitator training; and (e) have agreement from CEO, Chair and key clinical staff to participate and share aggregated service level data during the study.

### The PWG Complex Intervention

PWG facilitators are recruited via the PHC service. The PHC services nominate two women leaders, ideally Aboriginal or Torres Strait Islander, to work as PWG facilitators. It is important to have at least two PWG facilitators trained in order to provide support and improve sustainability of the local PWG (for example, should one facilitator resign their position, or leave the area).

PWG membership eligibility extends to all female community members who have an interest in MCH and may include but is not limited to the following:

- Pregnant women
- Mothers
- Women who may be pregnant but not yet aware
- Elders who are not health professionals.

An existing or new community women's health and wellbeing group forms the foundation of the complex intervention. Establishment of a new PWG or refreshing the membership of an existing group (if needed) will be led by the PHC service and may involve community consultation, engaging with existing community groups and other community organizations. PWG facilitators attend a training program during the set-up phase of the study. The purpose of this training is to build relationships and communication strategies between PWG facilitators and investigators; to share learning about facilitating the PWG; to become familiar with MCH audit data; and engage in a cyclical process of planning and implementation of changes to improve MCH called the Remote Service Futures (RSF) framework.

The RSF framework guides the PWG intervention planning and implementation process (37). The RSF uses a semi-structured approach to identifying needs, planning interventions, and implementation by community people in partnership with health professionals with monitoring via a series of workshops (38). This approach has recently been tested in Australian rural communities in improving oral health care, and found to be feasible when used with skilled facilitation (38–40). Table 1 outlines the RSF framework activities important in the PWG intervention.

**Table 1:** RSF framework activities important in PWG intervention.

**Phase 1: Planning workshops**	**Phase 2: Implementation workshops**
Identification of PWG members	Workshop 1: Monitoring implementation of planned MCH improvements
Workshop 1: Sharing knowledge: What are some things that PWG members know about that affect the health of mums and bubs? What are the strengths and limitations of the maternal and child health (MCH) support services for this community?	Workshop 2: monitoring implementation of planned change
Workshop 2: Sharing knowledge of what is happening in the care and health of mothers and babies in the community—through national and local data, health service information, and other information regarding social issues (family support, access to adequate child care or housing, family issues).	Workshop 3: monitoring implementation of planned change
Workshop 3: Women share ideas on what might lead to MCH improvement. What can be done in the community to improve the health/care of Mums and Bubs? Share knowledge of what other Aboriginal and Torres Strait Islander communities have done in other relevant area.	Workshop 4: monitoring implementation of planned change
Workshop 4: Developing a community MCH improvement plan with prioritized items	Ongoing PWG activity as desired by community Ongoing implementation. Translation of outcomes

Following training, the facilitators return to their communities and consolidate or establish their PWG. The facilitators work collaboratively with PWG members on MCH improvements using the RSF framework. It is important for PWGs to link with existing community initiatives (for example, Health Action Teams, Domestic Violence support groups, breastfeeding peer-counselors). The role of women in these groups is to: share local knowledge and community perspectives, focus on building community expectations, encourage community ownership of services, engage in activism for high quality care, and plan and develop initiatives to improve MCH. MCH nursing and/or medical staff may also be included to build their own capacity to respond to community perspectives.

During the intervention, each PWG decides the exact timing and interval of the four planning workshops. These may be at approximately monthly intervals (completed in the first six months of the intervention, with the flexibility to accommodate significant community events). There is an ongoing relationship between the investigators and the PWGs so that technical support, for example, extra data, assistance in using audit data, or information about implementation of changes, can be provided if necessary. The type or frequency of technical support is recorded for each PWG and form part of the process evaluation of the PWG. PWG facilitators and members are informed that the MCH improvement plan needs to be cost neutral and within the remit of the health service and PWG/community to implement within a 12-month period. Three initiatives from the community MCH quality improvement plan will be prioritized for implementation, and these will be implemented and monitored through a series of four workshops over 8 months at intervals to suit the services (at approximately bimonthly intervals). Annual meetings will bring facilitators together to disseminate their experiences, share learning and enhance the knowledge base for groups to respond to local needs, issues and priorities. Strategies considered and implemented by groups will differ according to prioritized goals and what is contextually appropriate and might involve health service or community level initiatives. Examples from previous PWG interventions include patient-held records, service report cards, breastfeeding support groups and community education programs.

As part of the intervention, services are provided with AU$5,000 per year to support the study at their facility as they wish (for example, part compensation for staff time, producing health promotion resources in response to PWG group input). In addition, PWG facilitators receive a small amount of reimbursement (0.1Full Time Equivalent) during the intervention period to directly off-set time and contributions to the facilitation of the PWGs. Both of these are factored into the cost effectiveness analysis.

### Outcomes

**Figure 1:**
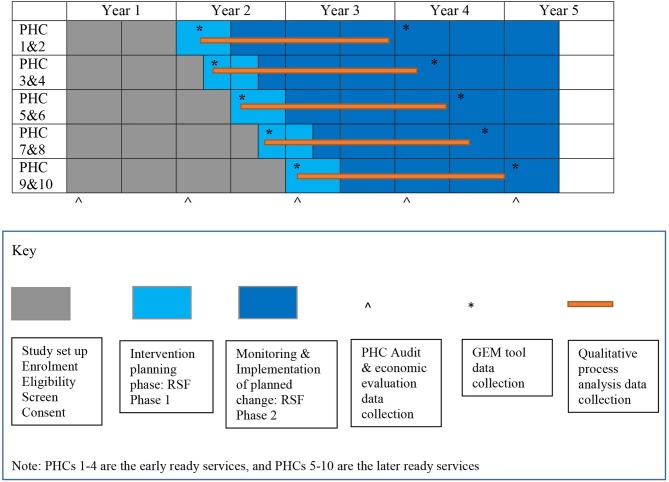
Stepped wedge design and participant timeline.

The primary outcome measure is a change in the MCH Quality of Care Index (QCI) from aggregated de-identified health service audit data. The MCH QCI provides an indicator for overall adherence to delivering recommended services based on evidence based clinical best practice guidelines in the delivery of care. The MCH-QCI (%) is calculated from audit data as (total number of services provided)/(total number of recommended services) ^*^100 (Table 2) (41). Baseline audit data is collected from services at yearly intervals (over ~1 week at the end of each year) for the 5-year duration of the study. Given the stepped wedge start-time of the intervention, each service will have at least one set of pre-intervention audit data (some up to three) and at least two sets of post-intervention audit data (some up to four; Figure 1). Patients attending the services whose charts may be audited are women with an infant aged between 2 and 14 months at the time of audit, resident in the community for at least 6 months of the pregnancy and using the PHC service as her usual source of care.

**Table 2:** Indicators included in MCH quality of care index.

Maternal Health QCI30 Clinical Indicators	Laboratory investigations: urinalysis (three time points), blood group, antibodies, full blood examination, rubella, Hepatitis B, syphilis serology, HIV, morphology US Physical examination: weight (two time points), body mass index; blood pressure (three time points); fundal height (two time points), fetal heart rate (two time points) History of risk factors: cigarette use, alcohol use, illicit drug use (each at two time points) Antenatal discussions: antenatal education, healthy living (nutrition, exercise, oral health, breastfeeding), psychosocial situation (domestic environment, family support, financial and housing)

Secondary outcomes: Secondary outcomes will include: (i) percentage of pregnant women having their first antenatal visit before 13 weeks gestation; and (ii) mean birthweight.

Process measures include changes in empowerment for PWG members [measured using the validated Growth and Empowerment Measure (GEM)] (22), alongside a theory-generating process evaluation using qualitative methodology informed by Indigenist and critical theory of the implementation and experience of the intervention (42, 43).

A cost-consequence analysis (CCA) and cost-effectiveness analysis (CEA) will be undertaken from the health system perspective. The outcomes for the CCA will be all primary and secondary outcomes for the study. The outcome for the CEA will be a 100 g increase in mean birthweight (as the main direct health outcome measure).

### Participant Timeline

The non-randomized cluster stepped-wedge design involves staged implementation of the intervention with two services commencing the intervention planning period at three monthly intervals (Figure 1). Initially, the study was planned to be randomized in terms of the timing of commencement of the intervention, but it became apparent that some of the services were ready much earlier than their allotted start date, based on the pre-existence of a functional women's group. We considered stratified randomization, but ultimately came to the conclusion (with our service partners) that imposing a randomized order on a complex intervention with a presumed theoretical basis of empowerment was likely to be counterproductive in terms of relationship building/engagement with community members and was unpopular with staff at participating services. Thus, randomization of start order was abandoned, and time of commencement will be adjusted for statistically as part of the analysis.

The first phase of the study is the start-up phase and involves study set up, engagement of stakeholders, recruitment of services, recruitment of PWG facilitators, training and formation of PWGs. At the first service engagement meeting in late 2018, services were paired in terms of timing of the intervention for practical reasons based on geographic proximity, to improve feasibility of support (both from the project team and from facilitators at the partner site). The four “early ready” services will embark on the complex intervention first, at three month intervals. The remaining six services have agreed an order of implementation based on perceived readiness (Figure 1). On commencing the intervention, there is a 6-month intervention planning phase comprising the RSF Phase 1 planning workshops and between two and three years for the implementation of MCH improvement plans and monitoring implementation of planned changes (RSF Phase 2) depending on the time of entry.

### Sample Size

The study is conducted with ten services that have self-nominated and been engaged with MCH audits. The number of maternal records examined in a service at a single audit cycle ranges from <5 to >100, but is usually ~20. Actual mean of the MCH-QCI from 43 services previously involved with One21seventy MCH audits is 65.97% with standard deviation (SD) of 13.58% and a change of 5% is likely to be clinically significant. A stepped-wedge design with five steps, ten clusters and intracluster co-efficient of 0.15 and an average cluster size of 20 has a design effect of 0.40 (with only two baseline and one subsequent audit) (44). Using our expected sample size of 200 audit records from 10 services and a design effect of 0.4 with alpha = 0.05, an SD of 13.58, 99% power is achieved for detecting a 5% change in our primary outcome. This is a conservative estimate. This sample size also gives a statistically significant result if mean birthweight is 100 g higher after the intervention than in the pre-intervention state (e.g., 3.3 kg v 3.2 kg). For the proportion of pregnant women with their first antenatal visit prior to 13 weeks gestation, this study will produce a statistically significant result if the odds of a pregnant woman attending in the first trimester are 1.5 times higher in the PWG group than in the pre-intervention group.

### Recruitment

Recruitment to this study is at three levels: PHC service recruitment, PWG facilitators and recruitment of individuals into the PWG.

#### PHC Services

An information sheet about the study and an invitation for services to self-nominate has been circulated through peak bodies, state health partners, investigators' networks. Follow up by meetings to provide further information and check eligibility has occurred with interested services. Attention has been paid to maximize variability amongst services as well as enabling services to make an informed choice about participation, considering all factors. Maximizing variability assists in determining how the intervention might be transferable to different settings. Service consent to participate involves meetings with the PHC service staff to discuss the study and written consent from an Aboriginal Community Controlled Health service Board or CEO to participate.

#### PWG Facilitators

Individuals are invited to participate as PWG Facilitators via the PHC health service. Members of the investigation team discuss the study with nominated PWG facilitators and seek written consent.

#### PWG Members

Individuals are invited to participate in the study via the PHC service and PWG facilitators. The PHC service determines the appropriate approach to recruitment depending on the context but includes activities: local advertisement, community meetings, via other local community groups and stakeholders. Interested individuals are provided information about the study and assessed for eligibility. Verbal and written consent to participate is obtained by the PWG facilitators. Within existing PWG groups, individuals who choose not to be part of the study will continue to participate in group activities outside of the MCH planning and implementation process.

PHC service and PWG retention are promoted by inviting PHC representatives and PWG facilitators to be members of the project steering committee. Regular email updates and newsletters are available to PHC service staff and PWG facilitators. In addition, a closed facebook group will be established so that they can share information and progress with each other. PWG participant retention is promoted by flexibility to the needs of the group when organizing planning and evaluation meetings; sharing newsletters and other study communications where appropriate; providing reminders of upcoming meetings and regular feedback on topics discussed.

Given the thorough processes that the services and investigator team undergo once services are involved it is unlikely that a service would withdraw. However, there is a plan in place if a service can no longer participate involving maximizing possible data collection for that service and adjusting the analysis accordingly. There may be changes in personnel at the PHC service and amongst the PWG facilitators. Comprehensive refresher training is available in any aspect of the study should PHC staff or PWG facilitators require this, managed within the pragmatic nature of the trial. Participants in the PWG may withdraw from the study for any reason at any time. If they choose to withdraw this will not affect their relationship with the PHC service or the investigator team. If they withdraw from the study they can request that data is returned or destroyed.

### Allocation

Two health services each commence the intervention at 3-monthly steps, and as discussed above, randomizing the order of intervention implementation was abandoned for ethical and theoretical reasons. This is now a non-randomized trial, still with a stepped wedge design and cluster intervention. Although not universally accepted, there are precedents for this design in the literature (45) including suggestions for analysis to compensate for the lack of randomization (46). The team's belief is that in a design with three month steps, and an expected time frame of 12 months for maximal effect the detrimental effects of this in terms of potential allocation bias are likely to be outweighed by relational benefits in terms of control and implementation. Services act as their own controls, thus no blinding at service level is either feasible or necessary. Analysis adjusts for the time of commencement of the intervention.

### Data Collection, Management and Analysis

#### Primary and Secondary Outcomes

Cross-sectional data for the primary and secondary outcome measures are collected from services by service staff using the One21Seventy MCH audit tool (35) (from which the MCH-QCI can be obtained) based on evidence-based best practice guidelines about quality MCH care. Processes for conducting these audits and ensuring data quality are well described (3, 7, 41, 47). The MCH-QCI is calculated at the beginning of the study and on an annual basis using existing mechanisms and supports that are part of the inclusion criteria. At least five sets of audit data will be available for each service. This is adequate time to demonstrate significant impacts on quality of care and possibly some health outcomes such as birthweight. Patients attending the services whose charts may be audited are women with an infant aged between 2 and 14 months, resident in the community for at least 6 months of the pregnancy and using the PHC service as her usual source of care.

Data, from which the primary and secondary outcome measures (MCH-QCI) will be drawn, is the property of the PHC service and the service forwards de-identified data to the research team for analysis. This data is in electronic form using a template designed by the research team. This template includes the name and a full description of the variable. Members of the investigator team including the statistician are responsible for checking the templates sent by PHC services and responding to any data queries.

The unit of analysis for the primary outcome is the health service. For the primary analyses (for both primary and secondary outcomes), it is likely that the PWG will have an effect on outcomes after 12 months from the start of the intervention at each step. The outcomes will be analyzed using linear mixed effects models. The models will have a random effect for community, and fixed effects for the effects of time. Estimates of the effect of PWGs will be reported along with 95% confidence intervals. Secondary analyses may adjust for rurality, service size, number of audit cycles and governance structure (ACCHS or government service), and various sensitivity analyses may be undertaken. Statistical significance is set at *p* < 0.05 level to test the hypothesis that there was a significant change in each of the outcome measures from baseline score. Imputation analysis is used to account for the effect of missing data.

#### Process Measures Evaluation

PWG facilitators keep a journal to capture evidence about the process of implementation, such as the number of women attending the meeting and their timing, areas of discussion, key learnings, other community members and organizations involved. In addition, regular communication with project managers and through the closed facebook group will allow the facilitators to discuss any issues and gain support from others.

Empowerment of women who are members and facilitators of the PWGs in each community is measured at the onset of the process (at the start of the intervention planning phase in each service), and at the end of the intervention period. This is done through the administration of the GEM, both the Emotional Empowerment Scale (EES) and the 12 Scenarios to measure the empowerment process (22). In addition, satisfaction with the PWG process, implementation and experience of the intervention and outcomes are evaluated using semi-structured interviews at the completion of the study. Participants include PWG participants and facilitators, along with some health care providers and community women who have not been involved in the women's groups. Around 10 interviews will be performed for each participating community.

GEM tool data is collected in hard copy, entered electronically by a member of the investigator team and original data kept in a locked cabinet at the sponsor organization. Data entered is cross checked by another investigator to increase the accuracy of data entry and coding. Digitally recorded qualitative interview data is transcribed as word documents. The team has developed a protocol to manage the de-identification and cross checking of qualitative data with respondents. This includes the creation of a flow chart for qualitative data management, standardization of file naming conventions, agreed de-identification processes and cross checking with other members of the investigator team.

The process evaluation strategy is based on robust strategies for evaluating complex interventions, aiming to develop a programme theory for the intervention (48). For process evaluation measurement using the GEM tool, pre and post GEM scores are analyzed using bivariate descriptive statistics. Qualitative evaluation of the process and outcomes using detailed interviews and observation via facilitator journals will uncover how participatory groups and individuals are able to produce and sustain change in MCH care, neglected in previous studies of women's group interventions. Qualitative data (journals and interviews) are transcribed in full. Transcripts are entered into NVivo and abductive coding (49) will first group the findings and do a line by line coding under each question and then inductively identify themes from the data using constant comparison. Analysis of the qualitative data is led by Aboriginal and Torres Strait Islander investigators. The combined findings will be used to generate an explanatory theory about how and why the intervention works (or otherwise) in a variety of contexts. In doing this we will integrate qualitative themes derived from trial data with Aboriginal and Torres Strait Islander knowledge frameworks and concepts (50).

#### Economic Evaluation

A cost-consequence analysis (CCA) and cost-effectiveness analysis (CEA) will be undertaken from the health funders perspective. Costs for the CCA and the CEA will be obtained from the trial data, and include all training (including annual meetings), staff time and resource use costs to the health system for a hypothetical nation-wide roll-out of the program. The outcomes for the CCA will be all primary and secondary outcomes for the study. The outcome for the CEA will be change in mean birthweight (as the main direct health outcome measure). The analysis will identify the incremental cost-effectiveness ratio (ICER) of a nationwide roll-out of the WOMB intervention compared to standard care. A budget impact analysis, that captures the impacts of the intervention on expenditure of the Federal and state governments, and individuals over a 5 year time frame, will also be undertaken.

Data management procedures for the economic analysis will follow the same process as primary and secondary outcome data and will be cross checked in consultation with health economists on the investigator team.

#### Implications and Significance

Despite growing international evidence, this study is the first to formally implement and assess in a cluster stepped-wedge design using quantitative outcome measures related to health care delivery, empowerment and health outcomes, the effect of applying a community participation intervention (PWG) to improve the quality of Indigenous MCH care in Australia. It brings together best practice in participatory approaches to strengthening health care with the expertise and reach of an experienced research collaboration to allow community members and health care providers to engage with MCH data from their community and implement changes to improve quality of care. Importantly (and often missing from previous research), the evaluation involves a cost-effectiveness analysis and a strong qualitative evaluation to learn how and why various components of the intervention exert any effects in various contexts. The economic evaluation also provides decision makers with evidence regarding the likely costs or cost-savings associated with wider roll-out of the intervention being tested.

Study outcomes will include: (i) refinement of a novel and scalable methodology with significant potential to engage community members and health care providers in MCH service improvement; (ii) development of a programme theory about mechanisms whereby PWGs exert their outcomes; (iii) direct transfer of findings to other Indigenous PHC services and to state and federal policy makers through well-established networks of knowledge exchange and partnership. Through this work the authors anticipate new knowledge of how best to facilitate improved quality of MCH care in Indigenous PHC settings and how to engage community members in driving health care improvements– this, in turn, will contribute to better intermediate health outcomes for women and babies and thus health outcomes for Indigenous Australians.

### Ethics and Dissemination

#### Ethical Approval

Ethical approval has been obtained through the Human Research Committee of the Northern Territory Department of Health and Menzies School of Health Research (ethics approval number is HREC 2018-3076); and James Cook University Human Research Ethics Committee (H7441). Additional ethical approval was obtained from the Western Australian Aboriginal Health Ethics Council (890) and the Aboriginal Health and Medical Research Centre Ethics Committee (1439/18). All eligible participants are required to provide signed informed consent before taking part in the study.

#### Data Management

All study related information is stored securely at the sponsor organization. All participant information is stored in a locked cabinet at the sponsor organization. All data sets are allocated an identification number to maintain participant confidentiality. Only study team members who are involved in data analysis have access to data.

Raw data are kept in a locked cupboard in the Principal Investigator's Office, and electronic data are stored on a password-protected computer. After the study finishes, electronic data will be transferred to a CD-ROM and also stored in a secure database repository and password protected. Electronic data on any other computers will be deleted. Raw data will be retained for at least 5 and 15 years respectively in accordance with NHMRC and the Australian Code for the Responsible Conduct of Research guidelines (51).

Participating services are involved in all decisions about data usage. No reference will be made in oral or written reports which could identify participants or the services without their written consent. None of these data, collected as part of this research, will be re-used without explicit permission. Raw data will be transferred to investigators involved in data analysis in other universities in accordance with protocols to safeguard data integrity.

#### Governance

The Steering Committee of the Centre for Research Excellence in Integrated Quality Improvement (CRE-IQI) is the overarching collaborative structure within which WOMB sits and consists of a broader network of Indigenous primary health care stakeholders. The CRE-IQI Steering Committee provides advice where required on the implementation of the actions devised by the PWGs, including the measurement of intermediate health outcomes and identify opportunities for broader roll-out and scale-up of study learnings.

The WOMB Steering Committee is responsible for monitoring conduct of the implementation, data collection and reporting. Committee membership comprises investigators, participant PHC services, facilitators of the PWGs and other members to be determined by the group. This group comprises members from the sponsoring organization and has no competing interests. It also assists in facilitation between the PWGs and their respective PHCs to implement action for mums and bubs; review and endorse interim reports; devise a knowledge transfer plan in phase 1 of the study; and assists with knowledge transfer activities throughout the life of the study.

Any protocol modification will be documented with appropriate justification and approved by the investigator team and steering committee. The final version will be presented to the relevant Ethics Committees, if required.

As the potential for harm based on this trial of women's group activity is extremely low, no formal stopping guidelines have been proposed. Ongoing process evaluation will ensure that any unanticipated unhelpful outcomes are identified and can be addressed.

#### Dissemination and Data Sharing

This study will be conducted in partnership with the PWG in each community, thus, overall results will be available for the PWG through existing auditing systems in the PHC services and a face-to-face visit to each service to present and discuss their own results overall. Interim progress reports will be made available to the steering committee of the CRE-IQI and study steering committee. The de-identified results of the research overall, across the ten services, will be made available to participating services through webinars, videos, and a face-to-face meeting.

This study will generate evidence about the effectiveness and cost effectiveness of community participation that is translatable to PHC settings in other Indigenous communities. Dissemination of findings to other Indigenous PHC services and to state and federal policy makers will occur through well-established networks of knowledge exchange and partnership; particularly through the networks of the CRE-IQI and through policy briefs and academic publication.

#### Trial Status and Protocol Version

Recruitment underway: Began April 2018 (services) and October 2018 (facilitators) and expect completion of recruitment approximately March 2020.

**Table:** 

April 2018 Original Version 1	
May 2018 Version 1.1	Primary reason for amendment – Ethical review process NT Health Ethics committee requested amendments to inclusion criteria to include women who are pregnant. Ethics committee review requested clarification on risk mitigation processes for participants
May 2019 Version 1.2	Primary reason for amendment- Modification of trial design from randomized to non-randomized. Statements were added and modified to improve clarity of trial design, participant inclusion and exclusion criteria and statistical methods.

## Data Availability Statement

The datasets generated for this study are available on request to the corresponding author.

## Author Contributions

SL, CF-B, JT, YC-J, RB, JF, MP, VM, EC, RE, AE, MZ, KC, RP, MR-M, and JK initiated the study design, contributed to the development of the study, and application for funding. EC, HF, MZ, and AE provided expertise in trial design and biostatistics. AE is conducting the primary statistical analysis. EC and HF are conducting the economic analysis. KC led the development of the protocol. All authors contributed to refinement of the study protocol and approved the final manuscript.

### Conflict of Interest

The authors declare that the research was conducted in the absence of any commercial or financial relationships that could be construed as a potential conflict of interest.

## Abbreviations

ACCHS: Aboriginal Community Controlled Health Services
CCA: Cost-Consequence Analysis
CEA: Cost-Effectiveness Analysis
CQI: Continuous Quality Improvement
CRE-IQI: Centre for Research Excellence in Integrated Quality Improvement
EES: Emotional Empowerment Scale
FWB: Family Wellbeing Program
GEM: Global Empowerment Measure
ICER: Incremental Cost-Effectiveness Ratio
MCH: Maternal and Child Health
MCH QCI: Maternal and Child Health Quality of Care Index
PWG: Participatory Women's Groups
RSF: Remote Service Futures.
